# Aesthetic Surgery Tourism: An Opportunity or a Danger?

**DOI:** 10.1007/s00266-024-04117-8

**Published:** 2024-06-24

**Authors:** Valentina Budini, Chiara Zanettin, Tito Brambullo, Franco Bassetto, Vincenzo Vindigni

**Affiliations:** 1 Division of Plastic, Reconstructive, and Aesthetic Surgery, Padua University Hospital, Via Nicolò Giustiniani, 2, 35128 Padova, PD Italy; 2 Department of Neuroscience, University of Padua, Padua, Italy

**Keywords:** Surgical tourism, Aesthetic tourism, Complications management, Public health, Patient education 2

## Abstract

**Background:**

Medical and surgical tourism is a rapidly growing phenomenon in post-pandemic Europe. The exodus of patients abroad to perform surgery, especially cosmetic treatments, is spreading recently in industrialized countries. In the social media era, the ease of access to advertising about surgical procedures and their costs favors traveling. However, the information given is not always punctual, and there is often a lack of patient awareness about the risks related to the procedures.

**Methods:**

The objective of the manuscript is to investigate, through clinical examples, the path that a patient operated on in a non-European country must face once he returns home. From the availability and translation of clinical documentation to the problems encountered in revision surgeries.

**Results:**

Thirteen cases of surgical tourism, patients who presented to our department with surgical complications, are investigated. Adverse events occurring during medical treatment abroad raised medico-legal and appropriateness issues, as well as concerns regarding the follow-up of patients.

**Conclusions:**

The current literature confirmed the high complication rate: It affects individual patients and the native country's healthcare systems. Therefore, patients must learn more about the dangers of traveling abroad for surgery. Education initiatives in the patient's home country might help achieve this.

**Level of Evidence IV:**

This journal requires that authors assign a level of evidence to each article. For a full description of these Evidence-Based Medicine ratings, please refer to the Table of Contents or the online Instructions to Authors www.springer.com/00266.

## Introduction

Since the turn of the century, "surgical tourism" has gained popularity in wealthier countries, especially the USA [1]. It lies in the exodus of patients to foreign countries to perform surgical, cosmetic, or dental treatments. Among the various branches of medicine involved, aesthetic plastic surgery interventions stand out in terms of the number and importance of cases [2].

The appeal of less expensive procedures and reasonably priced travel and lodging are the principal causes that incentivize international cosmetic surgery tourism [3].

Aesthetic surgical tourism is, however, a practice that can lead to a disastrous outcome. The initial price reduction frequently outweighs any potential longer-term risks for the patient and invalidates the careful selection of a capable surgeon and facility [4]. The sterility of the surgical facilities is uncertain or under-regulated, and infection is usually the most frequent complication [5].

The significance of the phenomena arises from:

- Ever-increasing cases also in Europe [6].
- Social media resonance, with advertising that reaches a large potential audience [7].
- Inadequate preoperative counseling. It is typically nonexistent or provided by an office secretary via video chat, with insufficient discussion of potential issues [8].
- Costs resulting from the absence of surgical follow-up fall on the healthcare system of the home nation. Neglectful post-operative care frequently results in patients having to manage wounds on their own, which frequently results in needless hospital visits and raises the complications rate [9].

The following discussion aims to delve deeper into the dynamics, clinical and healthcare aspects, and costs of patients operated on abroad who have been treated at home for related complications, using our institution's experience. The most common problems and the controversial aspects of this phenomenon emerged through a comparison with the available literature. Finally, some strategies to reduce and resolve issues related to surgical tourism are considered.

## Material and Methods

Between January 2020 and January 2024, a single-center retrospective investigation of cosmetic tourism complications was conducted at the Plastic Surgery Department of a Public Hospital.

All consecutive patients treated at our institution with complications of cosmetic surgery performed abroad have been included. There were no exclusion criteria.

Each patient provided a written agreement to process their personal information for clinical and research purposes. The primary data source for the research was our clinic's database, which updates all patient data. Patients were interviewed afterward on the telephone for additional information. Data collected include patient demographic information, country of original cosmetic tourist operation, the operation performed and complications; blood tests, diagnostic investigations, and surgical interventions necessary for those patients who sustained major complications.

**Fig. 1:**
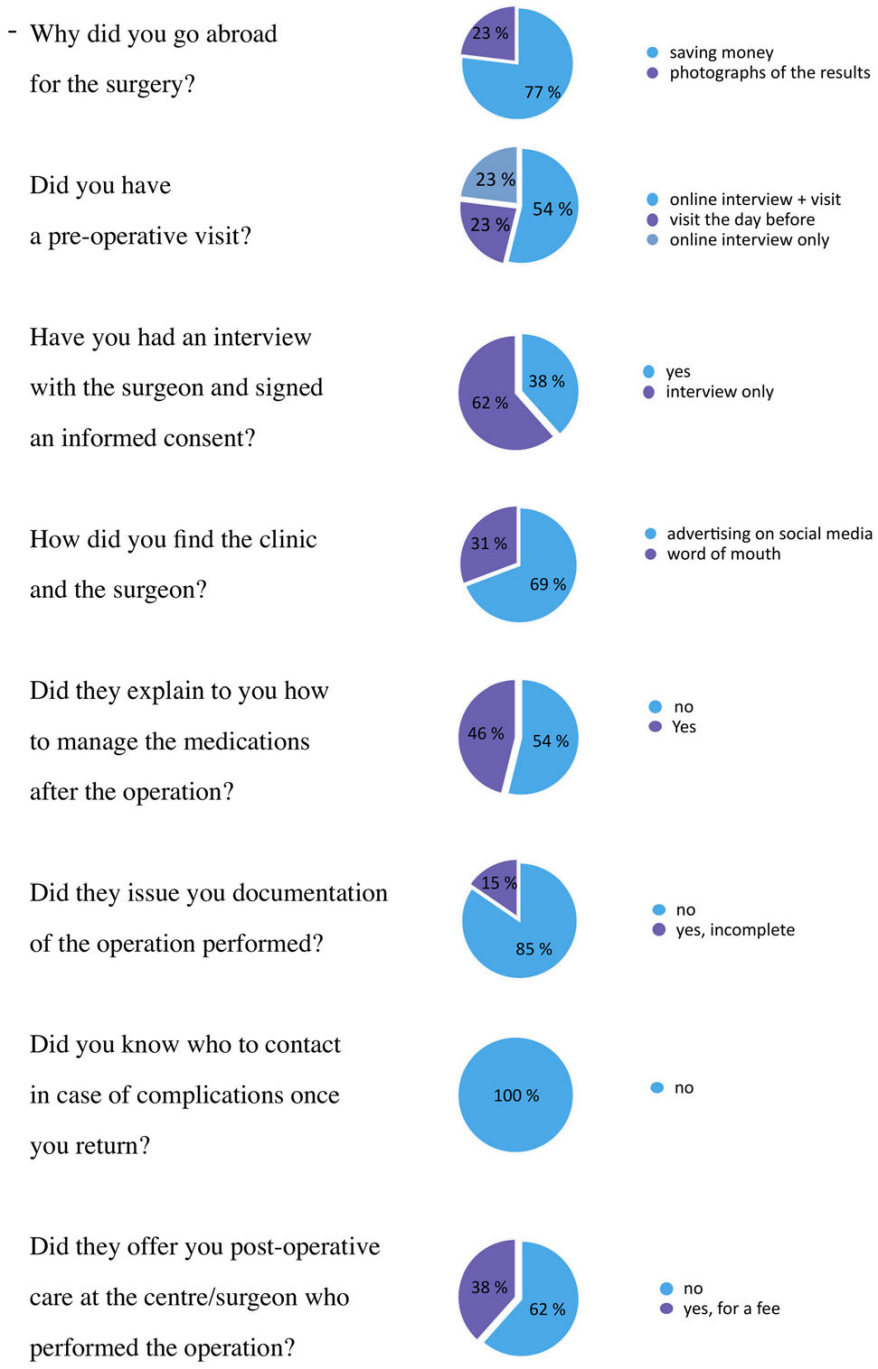
An anonymous questionnaire administered to the patients regarding their experience; each response is graphed as percentages

A specific anonymous questionnaire was also developed by our center and administered to each patient. Below are the eight questions the patients answered (Fig. 1):

- Why did you go abroad for the surgery?
- Did you have a preoperative visit?
- Have you had an interview with the surgeon and signed an informed consent that you understand
- How did you find the clinic and the surgeon?
- Did they explain to you how to manage the medications after the operation?
- Did they issue you documentation of the operation performed?
- Do you know whom to contact in case of complications once you return to your home country?
- Did they offer you post-operative care at the center/surgeon who operated?

## Results

Thirteen patients were treated for complications following cosmetic tourism. All patients were females. Ages ranged from 18 to 40 years (mean 28.8 years).

Three of the 13 patients were an everyday cigarette smoker, and 4 were an occasional cigarette smoker.

The mean body mass index (BMI) was 27.9 kg/m^2^ (range: 20-33 kg/m^2^). One of the patients had hypertension, and one of them had diabetes mellitus type II (under pharmacological control). No patient had a history of bleeding disorder, clotting disorder, or previous deep vein thrombosis or pulmonary embolism. One patient had already undergone breast augmentation surgery in her home country ten years earlier and was seeking revision surgery.

The most common operation performed was liposuction (*n *= 6), followed by abdominoplasty, breast augmentation (three each), and one case of thigh lift.

The most common operative country was Turkey (*n* = 7), followed by Albania (*n* = 5), and one patient underwent the procedure in Ghana.

Ten of the 13 patients initially presented to the Emergency Department (*n* = 77%). The most common complication was infection (*n* = 46%), followed by non-healing of the wound (*n *= 23%), discomfort in the management of dressings (*n* = 23%), and bleeding (*n* = 8%). Five of the 13 patients required surgery (*n* = 39%), and one required multiple surgeries to achieve complete healing.

Most cases of infection were following breast implant surgery (*n* = 66%). The bacteria found during the microbiological examination belonged to the species of normal skin colonizers or fecal bacteria. (*S. Aureus, Epidermidis; E. cloacae, E. Coli*) No atypical or multi-resistant bacteria were found, but intravenous antibiotics were administered in most of the cases of infection (*n *= 83%). With liposuction treatments, the most common consequence was discomfort with dressing and drainage management.

The mean outpatient follow-up was 16 weeks, ranging from 2 to 44 Weeks.

Nine of the 13 patients dropped out of the follow-up after being treated.

Figure 1 displays the responses to the survey that we conducted.

### Case Series

#### Case 1

A 32-year-old healthy woman who travels to Albania to get liposuction on her tummy, hips, gluteal area, breasts region, and arms.

On the third day following the procedure, the patient went to the ER after seeing a dip in one of the abdomen drainages. She underwent specialist consultation with patient education. The total cost of treatment, paid by public health, is 344 € (source: hospital discharge form).

#### Case 2

**Fig. 2:**
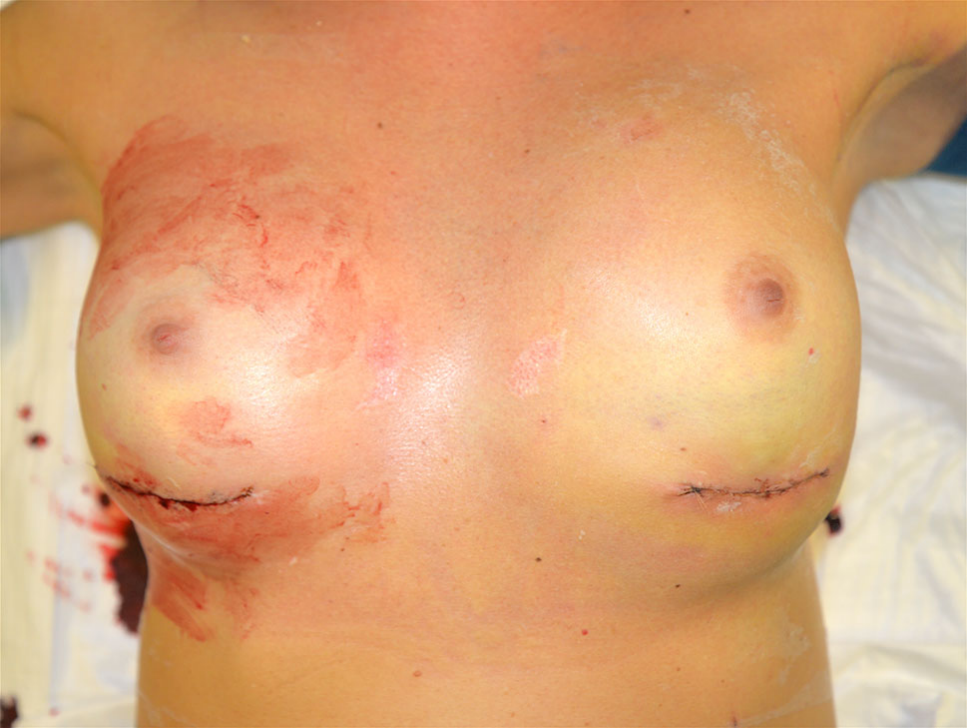
Bilateral hematoma complicating a breast augmentation surgery performed in Turkey

A 29-year-old healthy patient was scheduled to have bilateral breast augmentation surgery performed in Turkey. On the second post-operative day, she went to the emergency room due to bleeding from the surgical site in her right breast. She removed the bandage by herself (Fig. 2). Test results for blood revealed slight anemia and regular inflammatory indices. Bilateral hematoma was the ultrasound diagnosis.

Treatment was initially conservative, with broad-spectrum IV antibiotics. After further worsening, it was decided to hospitalize and drain the hematoma without the removal of prostheses and accurate hemostasis. The patient drops out her follow-up at our facility two weeks after discharge. The total cost of treatment, paid by public health, is 6415 € (source: hospital discharge form).

#### Case 3

**Fig. 3:**
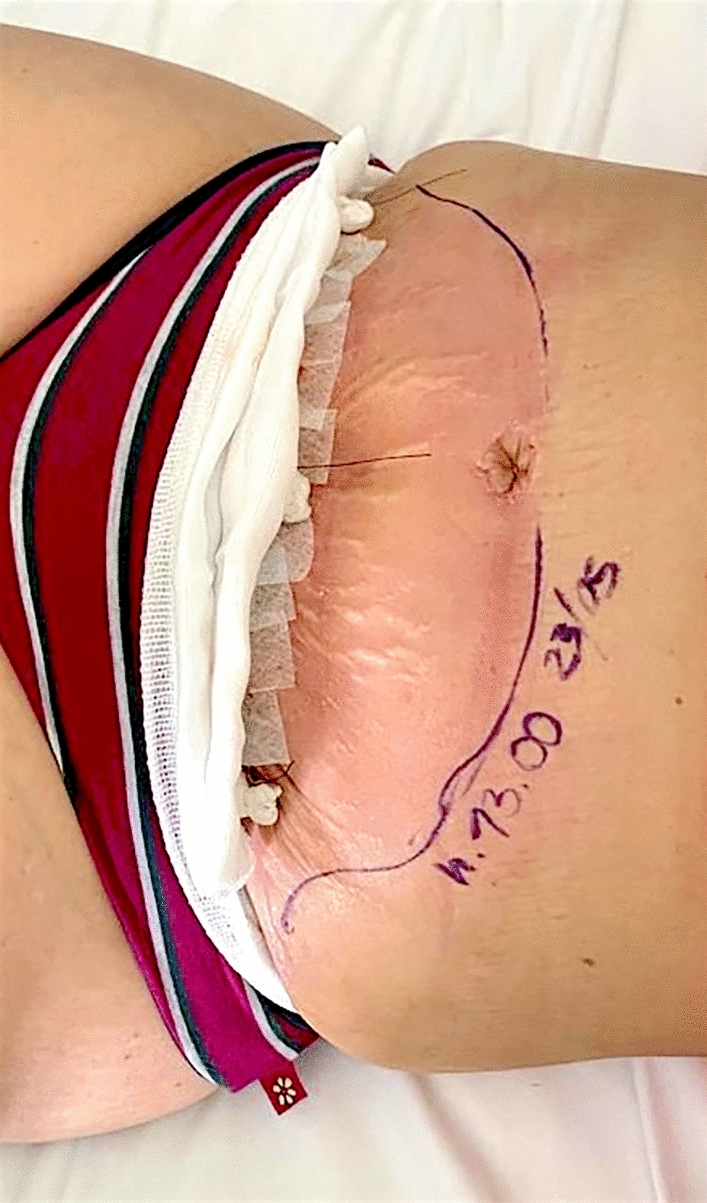
Necrotizing fasciitis after lipo-abdominoplasty performed in Turkey (8 day after surgery)

**Fig. 4:**
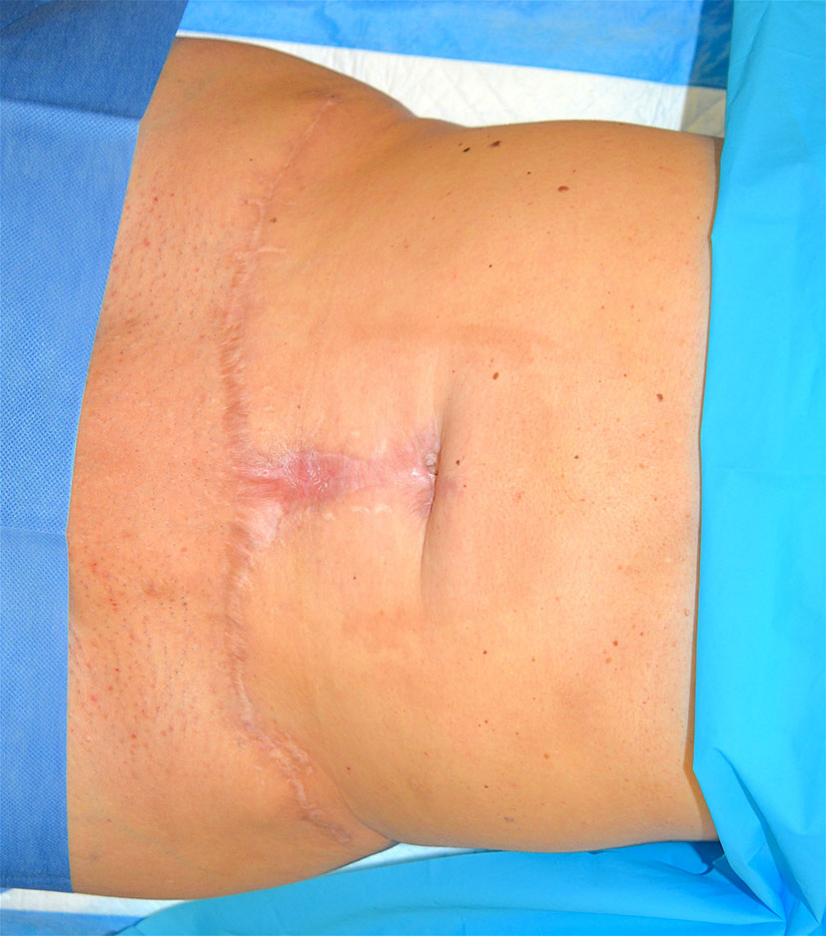
Same patient shown in Fig. 3, 9 months after resolution of the acute illness. Front view

**Fig. 5:**
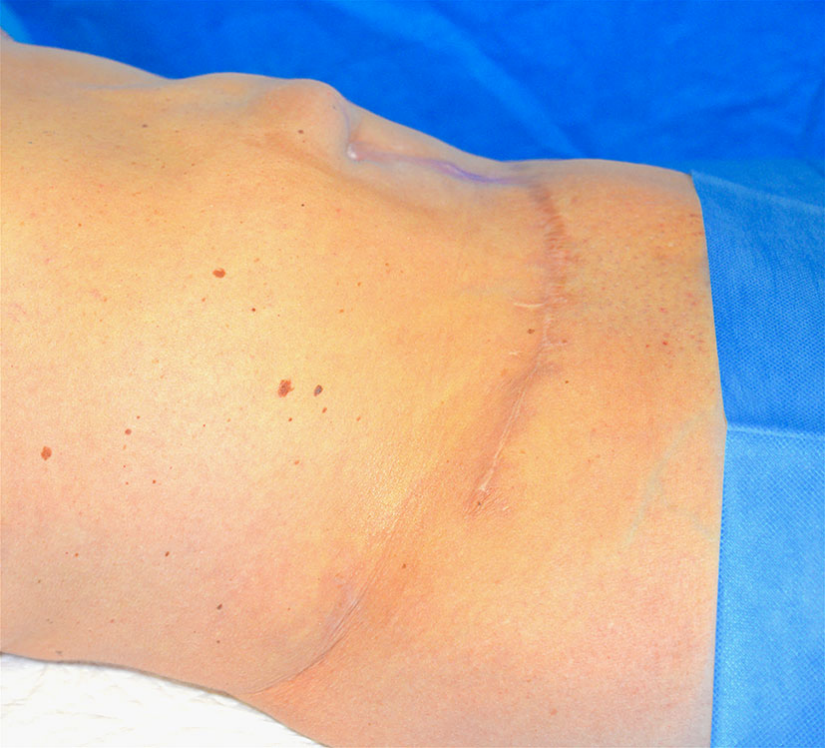
Same patient shown in Fig. 3, 9 months after resolution of the acute illness. Lateral view

**Fig. 6:**
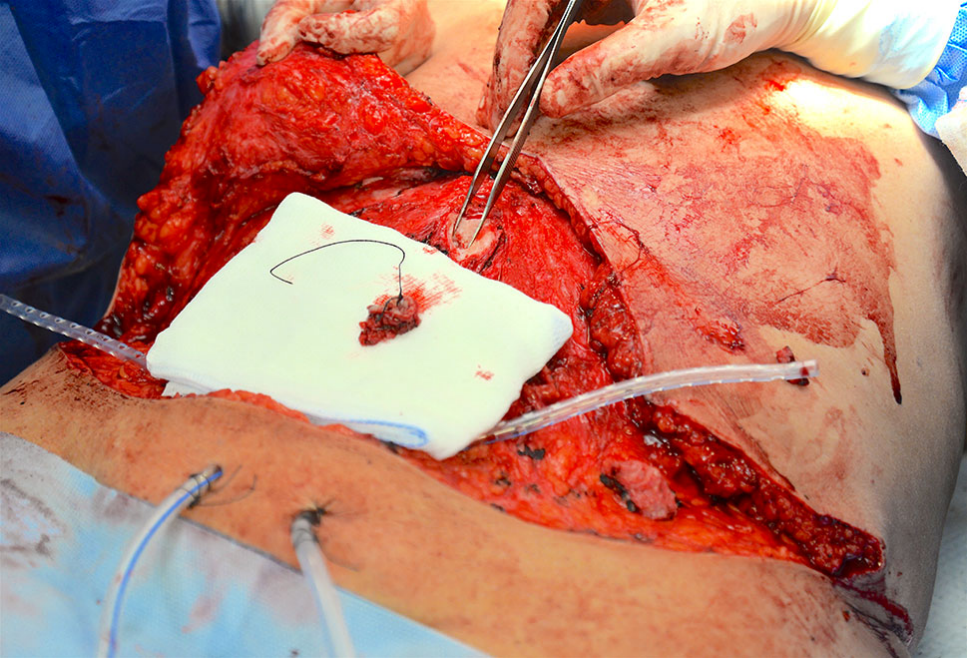
Same case shown in Figs. 3, 4, 5; during reconstructive surgery to reposition the navel and reduce epigastric bulging

A 37-year-old female patient affected by DM type II went to Turkey to perform abdominal liposuction surgery. Lipo-abdominoplasty was the final procedure, which the surgeon justified (without written consent) based on an unidentified abdominal hernia. We received the information about the surgery via audio in English, recorded and sent by the surgeon operator on the patient's phone. On the eighth day following the procedure, the patient went to the emergency room, claiming intense discomfort in the abdomen, erythema, tension in the region, and a fever that peaked at 39.7 °C (Fig. 3). Tests for blood chemistry revealed: WBC 17 × 10^9/L; Hb 11.2 PRC: 187 mg/L. The results of a CT scan found: "edematous imbibition surrounding tissues extending to the muscle fascia with the presence of gas bubbles, corpuscular material, and foreign body (suspected prosthesis mesh)." After being admitted to the hospital, the patient received intravenous broad-spectrum antibiotic therapy in addition to emergent surgical debridement. Following a microbiological analysis and cultivation of the collected material, the test result was positive for Enterococcus faecium. Two more reconstructive surgeries follow to obtain a complete healing (Figs. 4, 5, 6) The total cost of treatment, paid by public health, is 39991 € (source: hospital discharge form).

#### Case 4

**Fig. 7:**
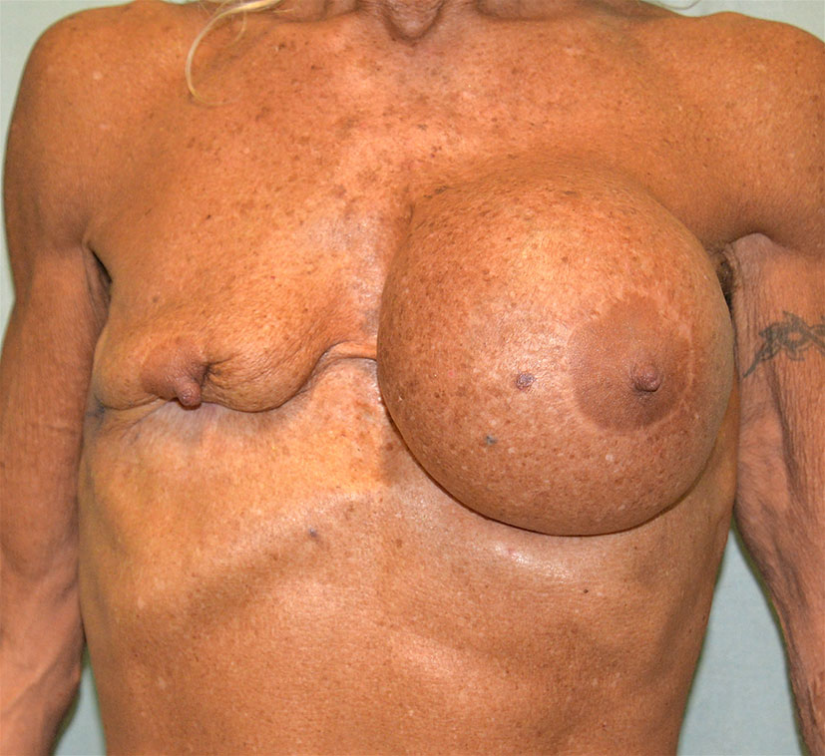
Extrusion of the right breast implant due to an infectious episode, after a surgery performed in Turkey. Fontal view

**Fig. 8:**
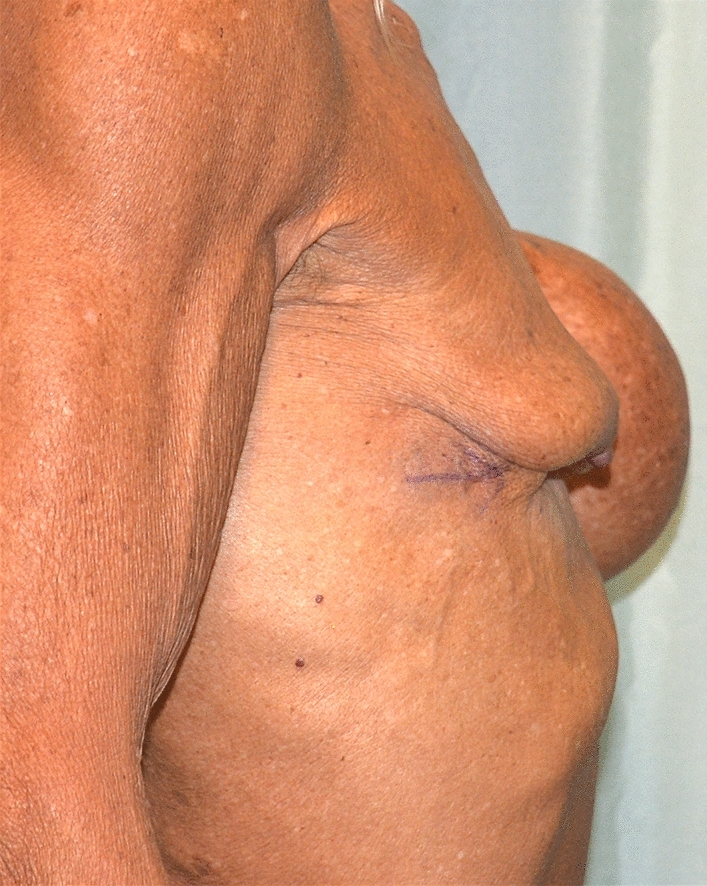
Extrusion of the right breast implant due to an infectious episode, after a surgery performed in Turkey. Lateral view

Extrusion of the right breast implant due to an infectious episode. Surgery performed in Turkey (Figs. 7, 8). The total cost of treatment, paid by public health, is 4827 € (source: hospital discharge form).

#### Case 5

**Fig. 9:**
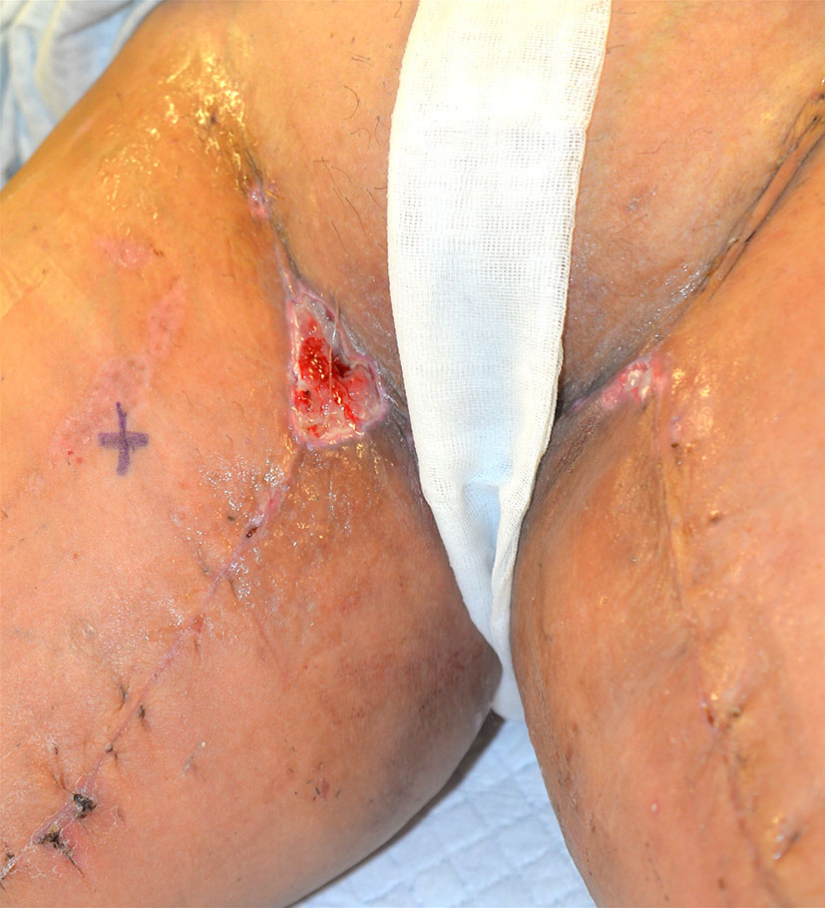
Wound dehiscence following thigh lift surgery performed in Albania

Wound dehiscence following thigh lift surgery performed in Albania (Fig. 9). The total cost of treatment, paid by public health, is 9840 € (source: hospital discharge form).

## Discussion

The number of plastic surgeon units managing complications from cosmetic surgery tourists has increased due to a rise in aesthetic surgery tourism.

In research assessing the public's perception of cosmetic surgery tourism among the UK-based general population, Nassab et al. discovered that 97% of respondents who had thought about undergoing cosmetic surgery would consider doing so abroad [10].

There is a lack of research regarding personal experiences dealing with complications resulting from cosmetic surgery tourism in Europe. In particular, there is a paucity of details regarding the cost, in terms of public health spending, of emergency room visits, follow-up visits, medical treatments, and revision surgeries that these patients undergo. Most examples in the literature come from overseas or from the United Kingdom [1].

Some current ASPS members were interviewed in the US in 2011 by Melendez and Alizedah on their experiences treating patients who traveled for cosmetic surgery. They claimed to have detected a rise in the patients' number bringing up issues related to cosmetic journeys. Most of the patients they treated experienced complications after breast augmentation or body contouring surgeries. Infection and dehiscence were the most frequent complications, followed by hematoma and irregular contours [11].

Miyagi et al. examined 19 cosmetic tourists with issues from 2007 to 2009 treated by a single plastic surgery practice in their observational study conducted outside of the UK. Among these, problems from breast surgery affected almost 75% of the patients; abdominoplasty followed. The two main complaints were dehiscence and wound infection. A facelift resulted in a marginal mandibular nerve injury for one patient, and an abdominoplasty caused a bowel perforation for another [12].

Klein et al. (Zurich, Switzerland) conducted a retrospective evaluation of their experience treating 109 patients who presented to their emergency department with complications resulting from cosmetic surgery tourism abroad in a 2017 study [4].

Patients who return from foreign countries after having performed aesthetic plastic surgery and present complications are a real economic and legal dilemma for the public health of the country of origin. It is made evident by cost-analysis research on cosmetic tourism issues published by Livingston et al. [13].

Often, the operative surgeon cannot be found or does not have an international broad certificate. The same happens with the clinic, which does not have a certification comparable to that of the European Community [14].

The possibility of even serious complications is present in every surgery. The real problem of surgical tourism is the impossibility of performing appropriate post-operative checks and a fair surgical follow-up. The caring relationship is usually lost as soon as patients are dismissed from the clinic, and there is frequently no further communication between the surgeon and the patient. As a result, the abandoned patient must handle any issues or complications on their own once they return home.

Our research displays that written documentation describing the operation is also incomplete, incomprehensible, or often absent. It poses a difficulty in understanding the cause and dynamics of the complication and in attributing legal responsibility for the complication. In the event of re-operation, there is also a risk that the entire responsibility for the outcome falls on the last surgeon and certified facility that operates on the patient.

Furthermore, for countries with public healthcare, there is no defined limit on the services or expenditures available in the event of serious complications. Deontologically, however, it is inconceivable to spend public money on a revision procedure that improves the aesthetic outcome of a cosmetic intervention made abroad [15].

Nowadays, there are still no specific guidelines - national or European—about how to manage these complications. Therefore, every nation and each center must face this lack, trying to calibrate its intervention by balancing the need for urgent intervention and the incompatibility of receiving treatment in a public hospital.

Our study is intended as a glimmer of awareness toward the surgical tourism topic and presents several limitations. The number of cases is limited. We have no data on the incidence or prevalence of the phenomenon as there is no national register of patients operated on abroad. It was not possible to carry out a case-control study as patients without issues do not reach the public hospital.

A targeted intervention, both in macroscopic governmental terms and in microscopic surgeon's clinical practice, should consider two fundamental elements: education and prevention. They are explicated into the following points:

- National or European guidelines for the management of complications
- Patient information regarding risks (which are involved in carrying out an operation in clinics not accredited by the Health System) and essential pre- and post-operative visits
- Regulation of medical advertising, by control of information content
- Emphasis on the importance and necessity of informed consent and written documentation

## Conclusions

From the retrospective analysis conducted in this study and among the available literature, we have found several critical issues in the constantly growing phenomenon of surgical tourism.

The low cost of the intervention, the attractive but non-instructive advertisements, the lack of punctual informed consent, and the emphasis placed on ancillary services (such as accommodation in luxury hotels included in the price) divert the patient's attention from the risks inherent in surgery and inhibit their ability to judge. Therefore, surgical tourism becomes, from a potential saving opportunity, a real risk for the patient's health and an expense for the public health system.

We hope that in the future, international and national organizations will create standards for managing the complications and will enforce regulation of medical advertising with more rigor. Improving the education of patients potentially at risk would also be desirable, emphasizing the importance of informed consent and written documentation of the surgery, in addition to the risks they run and the need for pre- and post-operative visits.

## Declarations

### Human or Animal Participants

This article does not contain any studies with human participants or animals performed by any of the authors.
